# The preservative polyquaternium-1 increases cytoxicity and NF-kappaB linked inflammation in human corneal epithelial cells

**Published:** 2012-05-06

**Authors:** Tuomas Paimela, Tuomas Ryhänen, Anu Kauppinen, Liisa Marttila, Antero Salminen, Kai Kaarniranta

**Affiliations:** 1 Department of Ophthalmology, Institute of Clinical Medicine, University of Eastern Finland, Kuopio, Finland; 2 Department of Ophthalmology, Kuopio University Hospital, Kuopio, Finland; 3 Department of Neurology, Institute of Clinical Medicine, University of Eastern Finland, Kuopio, Finland; 4 Department of Neurology, Kuopio University Hospital, Kuopio, Finland

## Abstract

**Purpose:**

In numerous clinical and experimental studies, preservatives present in eye drops have had detrimental effects on ocular epithelial cells. The aim of this study was to compare the cytotoxic and inflammatory effects of the preservative polyquaternium-1 (PQ-1) containing Travatan (travoprost 0.004%) and Systane Ultra eye drops with benzalkonium chloride (BAK) alone or BAK-preserved Xalatan (0.005% latanoprost) eye drops in HCE-2 human corneal epithelial cell culture.

**Methods:**

HCE-2 cells were exposed to the commercial eye drops Travatan, Systane Ultra, Xalatan, and the preservative BAK. Cell viability was determined using colorimetric MTT (3-(4,5-dimethyldiazol-2-yl)-2,5-diphenyltetrazolium bromide) assay and by release of lactate dehydrogenase (LDH). Induction of apoptosis was measured with a using a colorimetric caspase-3 assay kit. DNA binding of the nuclear factor kappa B (NF-κB) transcription factor, and productions of the proinflammatory cytokines, interleukins IL-6 and IL-8, were determined using an enzyme-linked immunosorbent assay (ELISA) method.

**Results:**

Cell viability, as measured by the MTT assay, declined by up to 50% after exposure to Travatan or Systane Ultra solutions which contain 0.001% PQ-1. BAK at 0.02% rather than at 0.001% concentration evoked total cell death signs on HCE-2 cells. In addition, cell membrane permeability, as measured by LDH release, was elevated by sixfold with Travatan and by a maximum threefold with Systane Ultra. Interestingly, Travatan and Systane Ultra activated NF-κB and elevated the secretion of inflammation markers IL-6 by 3 to eightfold and IL-8 by 1.5 to 3.5 fold, respectively, as analyzed with ELISA.

**Conclusions:**

Eye drops containing PQ-1 evoke cytotoxicity and enhance the NF-κB driven inflammation reaction in cultured HCE-2 cells. Our results indicate that these harmful effects of ocular solutions preserved with PQ-1 should be further evaluated in vitro and in vivo.

## Introduction

Benzalkonium chloride (BAK) is the most commonly used preservative in ophthalmic drops. BAK has cytotoxic effects and it has been shown to induce inflammation on the ocular surface cells in numerous in vitro and in vivo models [1-11]. Since the clinical treatment of glaucoma or dry eye syndrome usually requires a long-term topical drug therapy, ocular side effects may be potentiated by the use of preserved ocular drops [4,6,7,12]. Polyquaternium-1 0.001% (Polyquad^®^, PQ-1) is a detergent-type preservative derived from BAK. PQ-1 was formulated in the mid of 1980s by Alcon as a preservative for contact lens storage solutions. Nowadays, it is being increasingly used as a preservative in ophthalmic drops for glaucoma and artificial tear solutions. Recent findings reveal that PQ-1 has detrimental effects on cell membrane integrity and it induces cytotoxicity in ocular surface cells [13,14]. Cell membrane damage may activate inflammation and cytotoxicity via Toll-like receptors (TLRs) that are a class of proteins playing a key role in the innate immune system [15]. TLRs are classical inducers of nuclear factor kappa B (NF-κB) transcription factor that is a ubiquitous inducible transcription factor, and the master regulator of acute and chronic immune responses, cellular proliferation and cell death. The NF-κB protein complex is maintained in the cytoplasm in an inactive state by the presence of inhibitory kappa proteins (IκBs). In response to various stress stimuli, IκB kinase (IKK) can phosphorylate IκB proteins which, in turn, leads to NF-κB activation through the formation of a heterodimer of p50 and p65 NF-κB subunits which is then translocated to the nucleus where it triggers the activation of many inflammatory genes, such as interleukins IL-6 and IL-8 [15].

This study explored the hypothesis that PQ-1 might have some detrimental effects on ocular surface cells. Therefore, cytotoxicity, cellular permeability and the inflammatory effects of commercial eye drops containing PQ-1 0.001% (Travatan and Systane Ultra) or an eye drop with BAK 0.02% (Xalatan) and pure BAK 0.001% or 0.02% were analyzed using HCE-2 human corneal epithelial cell cultures.

## Methods

### Cell culturing and treatments

Cells used in this study were human corneal epithelial cells (HCE-2) obtained from American Type Culture Collection (ATCC, Manassas, VA). The cells were grown to confluency on 12-well plates (Cellstar^®^; Greiner Bio-One GmbH, Frickenhausen Germany) in a standard cell culture incubator (humidified CO_2_ 10% athmosphere and 37 °C). Keratinocyte- Serum Free Medium (SFM, with bovine pituitary extract and epidermal growth factor, cat. no. 17005–042; Life Technologies, Invitrogen, GIBCO®, Paisley, UK) containing insulin 0.005 mg/ml (cat. no. I-6634; Sigma-Aldrich, Steinheim, Germany), fetal bovine serum 10% (FBS, cat. no. CH30160.03; Thermo Scientific, Hyclone, Logan, UT) and penicillin 100U/ml + streptomycin 100 µg/ml (cat. no. DE17–602E; Lonza, Basel, Switzerland) was used as the culture medium. Fresh medium was supplied to the cells every other day and the cells were subcultured twice a week using 0.25% trypsin-EDTA (cat. no. 25200056; Life Technologies, Invitrogen) to detach the cells from plates.

The cells were exposed to the treatments (see below) for 5, 15, and 30 min, and then kept in the cell culture medium for 24 h, except for NF-κB activity test for 6 h, on 12 well plates before being analyzed. For every well 100,000 cells were seeded and cultured for 48 h before of exposures. Culture medium volume was 1 ml per dish, while for exposures used volume was 0.5 ml per dish. The cells were washed once with keratinocyte-SFM medium (without any supplements) before and after treatment to prevent any extra protein precipitation caused by BAK. The treatments were: 0-control (normal cell culture medium), Travatan (40 µg/ml travoprost, polyquaternium-1/polidronium chloride 0.001% as preservative; Alcon, Hünenberg, Switzerland), Systane Ultra (artificial tear drops, Polyquaternium-1/polidronium chloride 0.001% as preservative; Alcon), BAK 0.001% and 0.02% v/v aqueous solution (FeF Chemicals A/S, Køge, Denmark), and Xalatan (0.005% latanoprost, BAK 0.02% as preservative; Pfizer, New York City, NY).

### Cell viability

#### MTT assay

The cytotoxicity of exposure was measured with MTT-assay [16]. Color of MTT tetrazole salt was measured with a spectrophotometer at the wavelength of 570 nm. Briefly, fresh MTT solution (10 mg/ml in 1× PBS) was added (1:20 volume of medium) and the cells were incubated for 1.5 h. The cells were lysed and purple formazan dissolved into the solution by overnight incubation with MTT-lysis buffer (20% SDS, 50% N,N-dimethylformamide, 2% acetic acid, 25mM HCl; the volume of medium + volume of MTT-salt solution).

#### LDH assay

The permeability of cellular membranes following the exposures was determined by measuring the amount of released lactate dehydrogenase (LDH) enzyme from HCE-2 cells. The commercial CytoTox 96® -kit (cat. no. G1780; Promega, Fitchburg, WI) was used according to the manufacturer’s instructions. Maximum LDH release of HCE-2 cells was determined by lysing HCE-2 cells for 45 min (lysis buffer provided within the assay), and subsequently measuring the LDH from the culture medium. Absorbance values after the colorimetric reaction were measured at the wavelength of 490 nm with a reference wavelength of 655 a BIO-RAD Model 550 microplate reader (BIO-RAD, Hercules, CA).

#### Caspase-3

The levels of an apoptosis marker caspase-3 (active form) were measured from cell lysates using a colorimetric assay kit (cat. no. CASP-3-C; Sigma-Aldrich). Caspase-3 hydrolyses the peptide substrate Ac-DEVD-pNA (acetyl-Asp-Glu-Val-Asp p-nitroanilide) releasing pNA (p-nitroaniline) which can be measured at the wavelength of 405 nm. The assay was performed according to the instructions of the manufacturer. Caspase-3 (Product Code C5974; Sigma) was used as a positive control, and the Assay Buffer provided with the kit served as a negative control.The absorbance values were measured using a BIO-RAD Model 550 microplate reader (BIO-RAD).

### Inflammation

#### IL-6 and IL-8 assays

The concentrations (pg/ml) of IL-6 were measured from cell culture medium samples in duplicates by BD OptEIA™ Human IL-6 ELISA Set (cat. no. 555220; BD Biosciences, San Diego, CA). The assay was performed according to the instructions of manufacturer. For determining the IL-8 concentrations (pg/ml), the BD OptEIA™ Human IL-8 ELISA Set (cat. no. 555244; BD Biosciences) was used. The absorbance values after the colorimetric reaction were measured at the wavelength of 450 nm with a reference wavelength of 655 nm using a BIO-RAD Model 550 microplate reader (BIO-RAD).

#### NF-κB assay

NF-κB p65 ELISA kit (cat. no. EKS-446) was obtained from Enzo Life Sciences (Lausen, Switzerland) and used to measure p65 subunit after the binding to DNA. The cells were lysed in 25% glycerol, 0.42 M NaCl, 1.5 mM MgCl_2_, 0.2 mM EDTA, 20 mM HEPES before analyses, and the assay was performed according to the manufacturer’s instructions using 10 μg of protein per well. The luminescence signal was measured using VICTOR^™^ 1420 multilabel counter (PerkinElmer/Wallac, Turku, Finland).

### Statistical analysis

Statistical analyses were conducted with GraphPad Prism (Graphpad Software, San Diego, CA). Differences between groups were analyzed with the one-way ANOVA test followed by Dunnett’s post hoc tests. P-values below 0.05 were considered significant. There were six parallel samples in every analysis.

## Results

### Cellular viability

**Figure 1:**
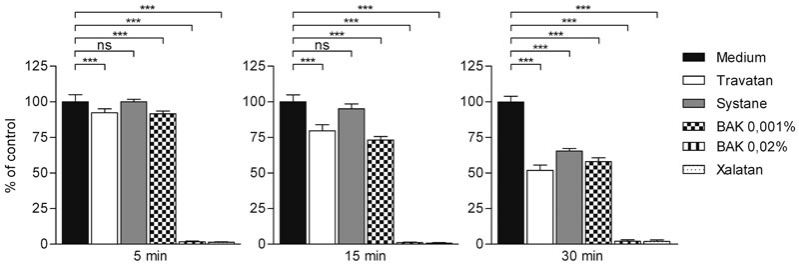
Level of cytotoxicity in HCE-2 cells analyzed by MTT assay. Columns represent the viability of cells (mean±SD. The viability of control cells is set as 100%. One-Way ANOVA, followed by Dunnett’s post hoc test, evaluated the statistical differences (n=6, *0.01<p≤0.05, **0.001<p≤0.01, ***p≤0.001, ns=not significant). Experiments were repeated three times.

MTT analysis revealed the extensive toxicity associated with BAK 0.02% containing Xalatan and BAK 0.02% (Figure 1). Both solutions evoked total cell death even after 5 min of exposure (followed by 24 h recovery in normal medium). At the same time point, the viability of cells exposed to Travatan, Systane Ultra and BAK 0.001% was near to the control. At later time points (15 and 30 min exposure + 24 h recovery) Travatan, Systane Ultra, and BAK 0.001% exposures reduced cellular viability in a time dependent manner. Based on the MTT analysis results and the extensive protein precipitation caused by the higher concentration of BAK (0.02%) and Xalatan, they were excluded from further experiments.

**Figure 2:**
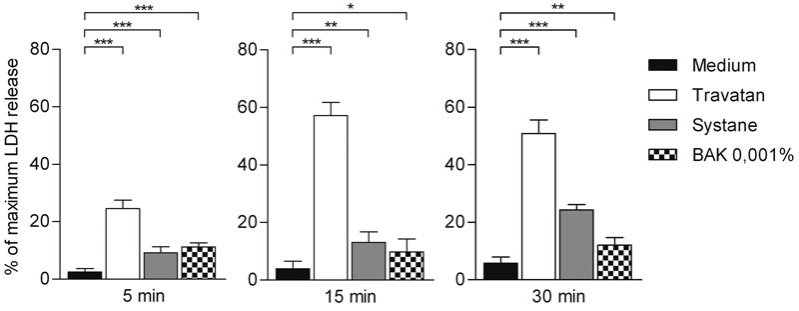
Released amount of lactate dehydrogenase (LDH) from HCE-2 cells. Columns represent the mortality (mean±SD) of cells, total cellular death being 100%. One-Way ANOVA, followed by Dunnett’s post hoc test, evaluated the statistical differences (n=6, *0.01<p≤0.05, **0.001<p≤0.01, ***p≤0.001, ns=not significant). Experiments were repeated three times.

Cellular permeability analysis, measured via the release of LDH, indicated that exposure to Travatan (preserved with PQ-1) was the most harmful (Figure 2). After 5 min exposure (followed by 24 h recovery in normal medium) the mortality was approx. 25%, which increased at later time points (15 and 30 min) up to 50%. In addition Systane Ultra and BAK (0.001%) were slightly toxic to HCE-2 cells as reflected in the decline in cellular viability from 10% to 20%. However, Systane Ultra seemed to be slightly more toxic at the 30 min time point.

**Figure 3:**
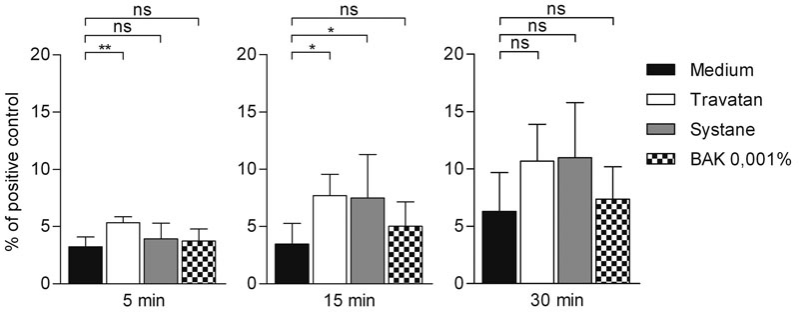
Active caspase-3 analysis in HCE-2 cells. Columns represent the proportion of caspase-3 from positive control which was set to be 100% (mean±SD). One-Way ANOVA, followed by Dunnett’s post hoc test, evaluated the statistical differences (n=6, *0.01<p≤0.05, **0.001<p≤0.01, ***p≤0.001, ns=not significant). Experiments were repeated three times.

Caspase-3 levels were elevated after 5 min of exposure to Travatan (followed by 24 h recovery in normal medium) when compared to control cells (Figure 3). Moreover, the exposure for 15 and 30 min to Travatan and Systane Ultra solutions increased the level of caspase-3, although the increase was not statistically significant.

### Inflammation

**Figure 4:**
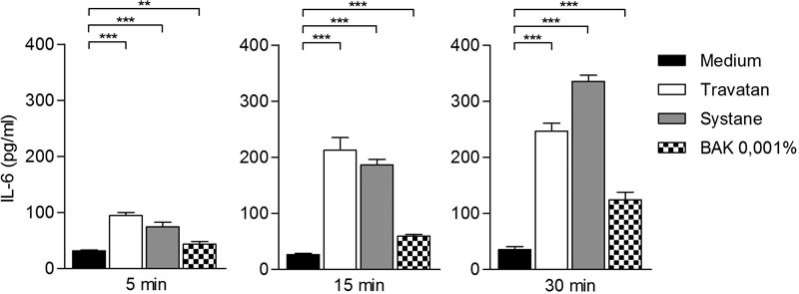
Interleukin-6 secretion from HCE-2 cells analyzed by ELISA. Columns represent the amount of IL-6 pg/ml (mean±SD). One-Way ANOVA, followed by Dunnett’s post hoc test, evaluated the statistical differences (n=6, *0.01<p≤0.05, **0.001<p≤0.01, ***p≤0.001, ns=not significant). Experiments were repeated three times.

**Figure 5:**
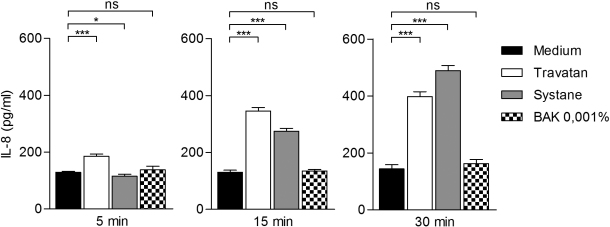
Interleukin-8 secretion from HCE-2 cells analyzed by ELISA. Columns represent the amount of IL-8 pg/ml (mean±SD).. One-Way ANOVA, followed by Dunnett’s post hoc test, evaluated the statistical differences (n=6, *0.01<p≤0.05, **0.001<p≤0.01, ***p≤0.001, ns=not significant). Experiments were repeated three times.

**Figure 6:**
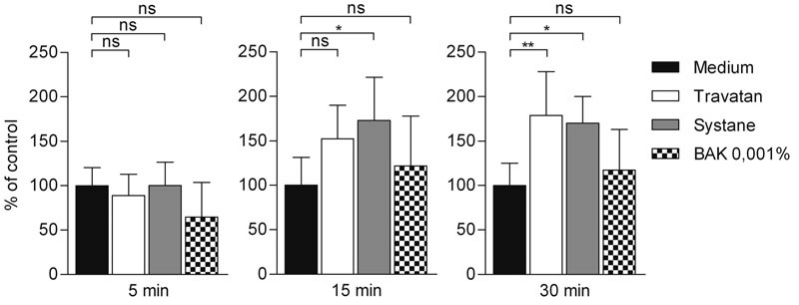
Binding of NF-κB (p65) to DNA 6 h after stimulation in HCE-2 cells. Columns represent the amount of NF-κB (mean±SD), control is set as 100%. One-Way ANOVA, followed by Dunnett’s post hoc test, evaluated the statistical differences (n=6, *0.01<p≤0.05, **0.001<p≤0.01, ***p≤0.001, ns=not significant). Experiments were repeated three times.

Travatan, Systane Ultra and BAK 0.001% increased IL-6 levels already after a 5 min exposure when compared to the controls (Figure 4). There was approximately three times more IL-6 released into the culture medium of the Travatan and Systane Ultra-treated cells. After 15 min of exposure to Travatan and Systane Ultra, the levels were 8 times higher than the controls. For comparison, BAK 0.001% increased the amount of IL-6 by only about twofold in response to 15 min´ treatment. The 30 min exposure amplified the levels even more in Systane Ultra (~10×) and in BAK 0.001%-treated (~4×) samples, whereas in Travatan exposed samples the IL-6 levels were the same as in the 15 min samples. The response of interleukin-8 was similar to that seen with IL-6 (Figure 5), although the elevations were not as dramatic (with Travatan ~50%–300%, Systane Ultra ~0%–350% and no elevation with BAK 0.001%). NF-κB levels were increased statistically significantly in the Systane Ultra (15 and 30 min exposures) and Travatan (30 min exposure) treated cells (Figure 6). BAK 0.001% did not have any effect on the NF-κB expression.

## Discussion

The ocular toxicity associated with BAK has been known for decades [17]. Preservative-free ophthalmic compounds are safer and better tolerated than BAK-preserved solutions [18], but antimicrobial preservatives are required for the use of multi-dosage medicine containers. New preservatives, such as polyquaternium-1 (PQ-1), have been developed as alternative to BAK [19]. PQ-1, a detergent-type preservative derived from BAK, has been used in artificial tears since the 1980s, and recent studies have assessed its suitability as a preservative for prostaglandin analogs used in antiglaucoma therapy [20]. The main disadvantage associated with PQ-1 is its tendency to reduce the density of conjunctival goblet cells, thereby decreasing the aqueous tear film production [21]. Despite being less toxic to the corneal surface than BAK, PQ-1 is known to induce damage to corneal epithelial cells as well [22]. This is the first study to demonstrate that while PQ-1 induces cytotoxicity, it also induces inflammation in an NF-kB-dependent manner in corneal epithelial cells.

Not only preservatives, drugs and excipients also may evoke potential cytotoxicity [23]. Several in vitro studies have shown that active antiglaucoma compounds do not exert cytotoxic effects [24,25]. On the contrary, recent studies have reported controversial results of protective effects of prostaglandin analogs against BAK-induced toxicity in human conjuctival cells [26-28]. The discrepancies in these studies might be due to the different BAK concentrations used as well as different cell line characteristics [22]. The drug solutions containing PQ-1 as preservative as well as BAK 0,001% showed cytotoxicity in a time-dependent manner in corneal epithelial cells, measured via the MTT assay, whereas the LDH assay revealed that exposure to Travatan (which contains PQ-1) evoked the most prominent LDH release of these three exposures as compared non-treated cells. The observed LDH increase may be a result of the drug i.e., travoprost, changing cell membrane integrity and function [23]. However, it is important to notice that LDH release does not always mean increased cytotoxicity, when analyzed by sensitive ELISAs.

Our findings with human corneal epithelial cells are in agreement with Brasnu et al. [23] who reported similar toxicity in conjunctiva-derived epithelial cells after 15 min of exposure to BAK 0.001%. However, in the present study it was found that PQ-1 0.001% containing eye drops showed similar or higher cytotoxic effects as BAK 0.001% after 15 min in cytotoxicity assays. The hypothesis of reduced cellular toxicity of PQ-1 is based on its larger size (approximately 27 times larger than BAK molecule) which results in its diminished capability of passing into the cornea [22,29]. In addition, PQ-1 lacks the hydrophobic domain present in the BAK molecule [22]. In earlier studies, the reduced cytotoxic effects of PQ-1 in comparison to BAK were explained by the latter’s corneal permeability [30]. Contrary to earlier results, the results from our study suggest that PQ-1 exerts almost as extensive cytotoxicity as BAK, in a time dependent manner i.e., depending on the cytotoxicity assay being used.

In vitro study does not mimic precisely the in vivo conditions; in vivo tears may provide additional protection as well as diminishing the direct contact time of cytotoxic agents to corneal cells. However, it has been proposed that detergent-type agents, such as BAK, can be absorbed into ocular tissues i.e., they can exert effects long after contact with the preservative has ceased [27,28]. Low concentrations (0.001%) of BAK can induce growth arrest and apoptosis in conjunctival cells in vitro for many hours after exposure to the compound [31]. In addition, BAK has induced necrosis when present at moderate concentrations, although apoptosis seems to be triggered low detergent concentrations (0.0001% to 0.001%) in a dose-dependent manner [31]. In a rabbit model it was reported that the cell-permeable preservative BAK can accumulate in the conjunctiva and cornea of the eye, and may therefore continue to act long after the actual drug is no longer present in tears [28,32]. It is not known whether PQ-1 exerts, at least in part, the same prolonged effects. However, the increase in caspase-3 activity induced by the PQ-1-containing eye drops after 15 min incubation was also observed at the other two time points although these increases did not reach statistical significance. Interestingly, BAK 0.001% did not affect the caspase-3 levels. The 5, 15, and 30 min incubations used in our study may mimic the innate contact of corneal exposure as well as in multi-treatment conditions, and these exposure times are in concordance with the in vitro schedules used in cytotoxic preservative corneal research.

The apoptosis and inflammation pathways are closely linked through their common mediators and transduction signals. It is a generally established dogma that apoptosis does not induce inflammation [33]. However, some pro-inflammatory cytokines, such as tumor necrosis factor alpha (TNF-α), interferon gamma (IFN- γ), and IL-1, can induce apoptosis [34,35] while IL-6 and IL-8 have been shown to inhibit apoptosis through several mechanisms [36,37]. The rapid induction of *IL-6* mRNA expression detected in corneal tissues is evidence that IL-6 is closely involved in the host corneal response during inflammatory conditions [38]. Our findings revealed that Travatan and Systane Ultra (which both have PQ-1) were able to induce the expression of IL-6 and IL-8 but not IL-1beta (data not shown). BAK 0.001% slightly increased IL-6, but had no effect on the IL-8 level. We observed a trend toward a caspase-3 (apoptosis marker) increase in a time-dependent manner with Travatan and Systane Ultra. In addition, we found a statistically significant NF-kB induction by eye drops containing PQ-1, indicating that the increase in IL-6 and IL-8 expression was being mediated via NF-kB activation. These data suggest that PQ-1 induces inflammatory rather than caspase-3 -mediated apoptosis in corneal epithelial cells and this mechanism is associated with activation of NF-kB.

In conclusion, the cationic polymer polyquad (PQ-1 0.001%) preservative was less cytotoxic in vitro than the highest commercial BAK (0.02%) concentrations. However, PQ-1 containing eye drops did induce even stronger inflammatory response than did BAK 0.001% exposure in human corneal epithelial cells. Further studies will be needed to elucidate the exact mechanism of this action, and whether these effects occur also in vivo, especially in conjunction with long-lasting therapy.
